# Public health round-up

**DOI:** 10.2471/BLT.22.010322

**Published:** 2022-03-01

**Ramping up COVID-19 vaccination in Africa**

A woman receives a COVID-19 vaccine at a vaccination centre in Paz Flor mall in Luanda, Angola. In January 2022, 96 million COVID-19 vaccine doses were shipped to Africa, more than double the volume shipped six months earlier. The vaccination rate needs to increase six-fold to meet the 70% population coverage target set for the middle of this year.

**Figure:**
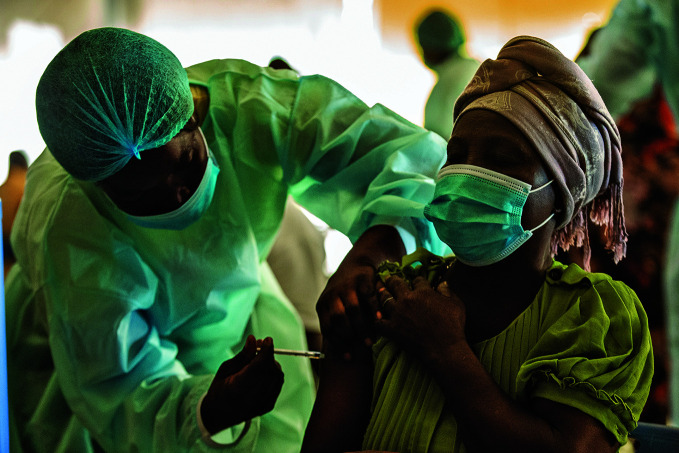
UNICEF/Carlos Cesar

## Afghanistan hit by multiple crises

A 5.2 magnitude earthquake hit the Qadis district of Badghis province, Afghanistan on 17 January and a landslide hit the Shahrak district of Ghor province on 19 January, adding to public health challenges that include a countrywide food crisis and multiple outbreaks of coronavirus disease 2019 (COVID-19), measles, acute watery diarrhoea, dengue fever and malaria.

The accumulated impact of these crises is overwhelming a health system on the brink of collapse due to lack of funding and resources. As of 31 January, only health facilities with external support were functional. The World Health Organization (WHO) was working with partners to deliver health services, including emergency services for the people affected by the earthquake and landslide. In addition, trauma and post-trauma physical rehabilitation services were being provided to 15 199 people through WHO-supported trauma care units and physical rehabilitation centres.

https://bit.ly/3HJWpQu

## COVID-19 vaccination in Africa

WHO, the United Nations Children’s Fund, the International Federation of Red Cross and Red Crescent Societies and partners launched an initiative to boost COVID-19 vaccine delivery to Africa.

As of 3 February, Africa had received more than 587 million COVID-19 vaccine doses – 58% through the COVAX Facility, 36% from bilateral arrangements and 6% through the Africa Vaccines Acquisition Trust of the African Union.

In January, 96 million doses were shipped to Africa, which was more than double the volume shipped six months ago. However, with only 11% (150 million of 1.4 billion) of the population fully vaccinated, the volume needs to increase six-fold if the continent is to meet the 70% coverage target set for the middle of 2022.

To support vaccine deployment, 50 technical experts had been deployed to 20 African countries by 3 February. Working under the leadership of the ministries of health, the experts are strengthening partner coordination, logistical and financial planning, management of data on vaccination uptake and vaccine stock, and surveillance of adverse events following immunization. They are also working with communities to strengthen trust in vaccination.

https://bit.ly/3gqnKLm

## Prequalification of first COVID-19 monoclonal antibody treatment

WHO added the first monoclonal antibody to its list of prequalified treatments for COVID-19 on 11 February. Called tocilizumab, the drug acts as an anti-inflammatory and to date has been authorized mostly for the treatment of arthritis. Given intravenously, it has been shown to reduce the mortality rate in patients who are severely ill with COVID-19.

Despite the patent for tocilizumab having expired for most of its uses, there is low global availability of quality-assured biosimilar versions of the drug. Prices for tocilizumab made by the originator company are high even in lower-income markets where prices around US$ 500–600 per single dose are reported.

It is hoped that companies producing biosimilars will now come forward to seek WHO prequalification, increasing the number of quality-assured products on the market and driving down prices. The prequalification of these products will also facilitate low- and middle-income countries’ authorization of them as COVID-19 treatments.

https://bit.ly/3p6OZ2v

## COVID-19 waste

Tens of thousands of tonnes of extra medical waste from the response to the COVID-19 pandemic is straining health-care waste management systems worldwide.

A new WHO report bases its estimate of the size of the problem on the approximately 87 000 tonnes of personal protective equipment procured between March 2020 and November 2021 and shipped to support countries’ COVID-19 response needs through a joint United Nations emergency initiative.

The authors of the report point out that other sources of waste include over 140 million test kits, with a potential to generate 2600 tonnes of non-infectious waste (mainly plastic) and 731 000 litres of chemical waste. The 8 billion doses of vaccine that have been administered globally have produced an estimated 143 tonnes of additional waste in the form of syringes, needles and safety boxes.

https://bit.ly/3otQjMg

## Microplastics in cigarette filters

The United Nations Environment Programme launched a partnership with the Secretariat of the World Health Organization Framework Convention on Tobacco Control to raise awareness and drive action on the extensive environmental and human health impacts of microplastics in cigarette filters.

Every year, the tobacco industry produces six trillion cigarettes, containing filters mainly composed of microplastics known as cellulose acetate fibres. When improperly disposed of, cigarette filters decompose releasing microplastics, heavy metals and many other chemicals.

Cigarette butts are the most discarded waste item worldwide, accounting for approximately 766.6 million kilograms of toxic trash each year. They are also the most common plastic litter on beaches, exposing marine ecosystems to microplastic leakages.

https://bit.ly/3HC4t5C

## Funding the ACT-Accelerator

The Access to COVID-19 Tools (ACT) Accelerator resource mobilization group agreed on a new financing framework designed to help end the COVID-19 pandemic as a global emergency in 2022.

The multi-agency ACT-Accelerator initiative provides low- and middle-income countries with the tests, treatments, vaccines and personal protective equipment needed to respond to the pandemic.

With a significant proportion of the global population still unable to get vaccinated, tested or treated, US$ 16 billion in grant funding is urgently required from governments to fund the work of the ACT-Accelerator agencies.

The framework agreed on by the ACT-Accelerator Facilitation Council’s Finance and Resource Mobilization Working Group sets out guidance on the “fair share” of financing that richer countries should each contribute to the ACT-Accelerator’s global response. The shares are calculated based on the size of their national economy and what they would gain from a faster recovery of the global economy and trade.

On 9 February, world leaders called on the governments of higher-income countries to meet the US$ 16 billion ACT-Accelerator funding gap and US$ 6.8 billion in-country delivery costs to support efforts to end the pandemic.

https://bit.ly/3swB1aL

## Pandemic impacts essential health services

Two years into the COVID-19 pandemic, health systems continue to face significant challenges in providing essential health services. According to the latest WHO pulse survey which was released on 7 February, disruptions were reported in over 90% of countries for services across the spectrum of care.

In more than half of the 129 countries and territories surveyed, many people are still unable to access care at the primary care and community care levels. Significant disruptions were also reported in emergency care, with a third of countries reporting disruptions to ambulance and emergency room services and just under a quarter reporting disruptions to emergency surgeries. Disruptions to rehabilitative care and palliative care were also reported in around half of the countries surveyed.

https://bit.ly/333WRtw

**Cover photo**

A young girl shows her proof of having received the polio vaccine in Rawalpindi, Pakistan, where, despite a six-month pause due to the pandemic, health workers managed to vaccinate over 39 million children under five years of age.

**Figure:**
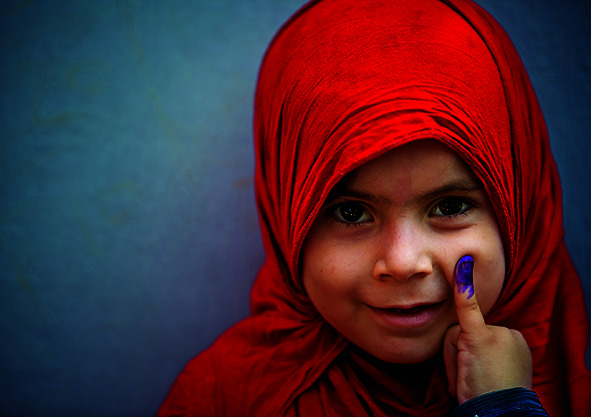
UNICEF/Syed Mehdi Bukhari

## Health-care providers and female genital mutilation

WHO and partners launched two new guides to help health-care providers care for girls and women who have been subjected to female genital mutilation.

Beyond healing physical harms, health-care providers can play a key role in addressing sequelae that include impacts on mental and sexual health while also influencing attitudes to help prevent new cases.

In recent years there has been an increase in so-called medicalization of female genital mutilation, in which health-care providers themselves carry out the procedure. Part of the intention of these guides is to help reverse that trend.

https://bit.ly/3sft5dO

## New ICD-11 released

The Eleventh Revision of the International Classification of Diseases (ICD-11) was released on 11 February. Compiled with input from over 90 countries and unprecedented involvement of health-care providers, ICD-11 is entirely digital, has a new user-friendly format and multilingual capabilities.

The ICD provides a common language that allows health professionals to share standardized information across the world. It is the foundation for identifying health trends and statistics worldwide, containing around 17 000 unique codes for injuries, diseases and causes of death. By using code combinations, more than 1.6 million clinical situations can now be coded.

https://bit.ly/3Jk7urL

**Looking ahead**

3 March. World Hearing Day 2022. https://bit.ly/3GEHvts

15 – March. Health for All Film Festival https://bit.ly/3utsKnp

17–18 March. High-level meeting on health and migration in the WHO European Region. https://bit.ly/3Des3T6

